# GNE myopathy: History, etiology, and treatment trials

**DOI:** 10.3389/fneur.2022.1002310

**Published:** 2022-10-18

**Authors:** Jeffrey Mullen, Khalid Alrasheed, Tahseen Mozaffar

**Affiliations:** ^1^Department of Neurology, School of Medicine, University of California, Irvine, Irvine, CA, United States; ^2^Pathology and Laboratory Medicine, School of Medicine, University of California, Irvine, Irvine, CA, United States; ^3^The Institute for Immunology, School of Medicine, University of California, Irvine, Irvine, CA, United States

**Keywords:** sialic acid, ManNAc, HIBM, GNE myopathy (GNEM), GNE gene, neuromuscular

## Abstract

GNE myopathy is an ultrarare muscle disease characterized by slowly progressive muscle weakness. Symptoms typically start in early adulthood, with weakness and atrophy in the tibialis anterior muscles and with slow progression over time, which largely spares the quadriceps muscles. Muscle biopsy shows atrophic fibers and rimmed vacuoles without inflammation. Inherited in an autosomal recessive manner, patients with GNE myopathy carry mutations in the GNE gene which affect the sialic acid synthesis pathway. Here, we look at the history and clinical aspects of GNE myopathy, as well as focus on prior treatment trials and challenges and unmet needs related to this disorder.

## Introduction

GNE myopathy is a rare inherited muscle disease. Known by many different names (“Nonaka distal myopathy,” “distal myopathy with rimmed vacuoles,” “hereditary inclusion body myositis,” “quadriceps-sparing myopathy,” among others), this condition results in progressive muscle weakness resulting from changes in the bifunctional enzyme UDP-N-acetylglucosamine (GlcNAc) 2-epimerase/N-acetylmannosamine (ManNAc) kinase.

This disorder was first described in the early 1980s by Nonaka et al. in Japan, but since then has been seen throughout the world, often in clusters (1). GNE myopathy is rare, though recent evidence suggests that the disorder may be more prevalent than previously thought (2). Initial symptoms typically appear in the third decade of life, with distal leg weakness often affecting the ankle dorsiflexors and resulting in foot drop. This is followed by slowly progressive muscle weakness affecting arms and legs, with relative sparing of the quadriceps muscles. Workup may reveal normal or mildly increased creatine kinase (CK) level, and muscle biopsy may show rimmed vacuoles and 14–18 nm filamentous inclusions, without inflammatory infiltration. Diagnosis is confirmed with homozygous or compound heterozygous GNE gene mutations.

The estimated worldwide prevalence of GNE myopathy is 1–9 per million (3). Recent work demonstrated that 31% of patients with undiagnosed genetic myopathies in the Indian subcontinent were found to have pathogenic GNE mutations (2, 4). This was further demonstrated in a more recent assessment (5). An estimated 1 in 203 people worldwide are thought to carry a potentially pathogenic GNE mutation. Despite this, the number of reported cases of GNE myopathy worldwide remains only slightly over 1,000, which is likely much less than the actual number of cases. Previously this discrepancy may have been at least partly related to the many names associated with the disorder, and presently may be affected by factors such as access to genetic testing and differences in clinical features such as a lack of vacuoles on biopsy in many cases (6).

The GNE gene product is an enzyme which is critical in the production of sialic acid. Sialic acid is a monosaccharide which binds to glycoproteins and glycolipids with several functions including cellular recognition and adhesion (7). Therapeutic trials have focused on this pathway, with the goal of supplementing sialic acid levels.

## History and geographic variations

In 1981, Ikuya Nonaka et al. described three cases from two families with distal weakness “predominantly affecting the tibialis anterior muscles,” and muscle biopsy showing rimmed vacuoles containing “numerous concentric lamellar bodies” (1). In 1984, Zohar Argov described nine cases among four Iranian-Jewish families presenting with generalized weakness, where the “quadriceps muscle was the only leg muscle which retained its normal power” (8). The autosomal-recessive inheritance pattern was also characterized. In 1995, Mitrani-Rosenbaum et al. localized the implicated gene to chromosome 9p1-q1 (9). In 2001, this was further specified to mutations involving the GNE gene (10). As the separately described cases were found to have a similar genetic basis by 2002, it became evident that these disorders represented the same condition (11).

The genetic characterization of GNE myopathy has highlighted regional genotypes (Table 1). The Middle East, Japan, India, Britain, and other regions of the world have mutations common to their respective areas. In the Middle Eastern population for example, the p.M743T mutation is frequently seen, and is seen in the surrounding region consistent with migration history. All of the Israeli patients described by Argov were found to be homozygous for the p.M743T mutation (referred to as the p.M712T mutation in the previous nomenclature system) (19). Argov identified 129 patients from 55 families, all of which were homozygous for the p.M743T mutation. Likewise, the common mutation among those of Iranian-Jewish descent is the p.M743T mutation, suggesting a founder effect. This genotype is also common in nearby locations such as Uzbekistan, Afghanistan, and Iraq, likely due to local population expansion. This mutation is also frequently identified in California, United States due to the Iranian-Jewish population in this region resulting from migration since the early twentieth century. This mutation is also frequently identified in Iranians of non-Jewish descent along with the less common p.R277Q variant (22). The p.M743T mutation has also been described in Muslim patients in Tunisia (23).

**Table 1:** Common GNE myopathy variants and associated regions.

**Mutation**	**Ethnicity or region commonly reported**
p.C44S	Japanese (12)
p.D207V	Japanese, Chinese (11, 13)
p.V603L	Japanese (14)
p.L539S	Chinese (13)
p.I618T	Bulgarian Roma, Rajasthani region of India (2, 15, 16)
p.V727M	Indian (17, 18)
p.M743T	Middle Eastern (4, 19, 20)
p.D409Y	North British (21)
p.A662V	North British (21)

GNE mutations in other areas of the world also exhibit a founder effect. In the Bulgarian Roma population for example, the p.I618T mutation accounts for more than 99% of mutations causing GNE myopathy (15). Notably, this same mutation is prominent among the Rajasthani people in North West India. Khadilkar et al. assessed 26 GNE patients from northwestern India. In this analysis, all 7 patients of Rajasthani origin harbored the p.I618T mutation, the same mutation seen in the Bulgarian Roma, with 5 of those 7 being homozygous for this mutation (16). All of the patients from the state of Rajasthan were from the Maheshwari and Jain communities specifically. It is speculated that the Roma population of Europe originated from this region of northwestern India including from the state of Rajasthan, during a period of migration possibly between the fifth and tenth centuries. This theory is further supported by studies analyzing single nucleotide polymorphisms as well as mitochondrial gene sampling (24, 25).

Outside of the Rajasthan population of northwest India, the most prevalent GNE mutation in the Indian subcontinent is the p.V727M mutation. Nalini et al. found that patients from six out of the eight families studied were found to have the p.V727M mutation. This mutation was also found in each of the four patients from Thailand as described by Liewluck et al., suggesting a possible founder mutation in this region as well (26).

In the North of Britain including Northern England, Scotland, and North Ireland, Chaouch et al. identified two frequently recurring mutations in GNE patients in that region, the p.A662V, and the p.D409Y mutations. 18 of 20 identified GNE myopathy patients in this region carried at least one of these two mutations (21), and 10 of the 20 patients were compound heterozygotes for both mutations. In this cohort, the mutations characteristic of other Middle Eastern or Japanese populations were not seen.

In Japan, where GNE myopathy was first described, a number of different mutations are seen. In a 2014 analysis of 212 Japanese patients with GNE myopathy, 63 different mutations were observed. The most frequent mutation in the Japanese population is the p.V603L mutation, accounting for over 48% of total mutations (27). The second most common mutation in Japan is the p.D207V, which is considered to be a milder phenotype in contrast with the p.V603L mutation which may be more severe (27). Similar to other areas of the world, the variants characteristic in the Japanese population are also seen in neighboring regions in East Asia including China and Korea.

Though some mutations are considered to be more severe than others, the correlation between genotype and phenotype is not entirely clear. Some evidence suggests phenotypic similarity among patients with the same mutation. Several of the Bulgarian Roma patients described by Chamova et al. presented with hand weakness, though still the vast majority initially presented with ankle dorsiflexion weakness (15). In fact, despite nearly all of the patients being homozygous for the same mutation, there was variability in presentation including age of onset and presenting symptoms. The Northern Irish patients with the p.A662V mutation were reported to exhibit a gradient of weakness in the deep finger flexors with more involvement of the index finger in comparison to the little finger (21). A potential correlation has also been considered regarding whether the epimerase or kinase domain is affected. Homozygotes for mutations affecting the kinase domain (KD/KD genotype) in the Japanese cohort appeared to have a more severe course in comparison to compound heterozygotes with one of the mutations affecting the kinase domain and the other mutation affecting the epimerase domain (KD/ED genotype) (28). However, it should be noted that the majority of the kinase domain mutations were the p.V603L mutation, which is considered to be a more severe phenotype as noted above (13). A more recent analysis of 89 patients from many countries showed that patients with the KD/ED genotype reported an earlier loss of ambulation in comparison to the KD/KD or ED/ED genotypes, though this did not reach statistical significance (29).

Pogoryelova et al. reviewed GNE registry data and 11 articles including 759 patients in order to further investigate the potential genotype/phenotype correlation. It was estimated that genotype accounts for 20% of the phenotypic variability seen in GNE myopathy (30). Several phenotypic trends were seen among patients with the same mutations. For example, patients harboring the p.L539S mutation appear to have an age of onset an average of 7.2 years earlier than those who do not have this mutation. The p.V603L seems to result in a lower probability of ambulation at age 40, but did not appear to affect the age of onset. It is also noted that some features that appear to be more prominent in some regions, such as the Beevor sign which has been described in the Indian cohort, may be attributable to other factors or observational bias.

However, the overall evidence remains unclear that genotype reliably influences phenotype. Every cohort described, despite sharing common mutations, shows variable presentations including weakness pattern and age of onset. It is also notable that the Middle Eastern cohort described by Argov included five clinically unaffected subjects despite homozygosity for the prevalent mutation even at 50 and 68 years of age suggesting incomplete penetrance (4).

## Clinical manifestations

There are a number of clinical features which are commonly seen in GNE myopathy (Table 2). Symptoms most frequently appear in the third decade of life, though cases have been reported with onset as early as age 10 and as late as 61 years of age (31). The majority of patients present with distal weakness in the legs, typically of the tibialis anterior muscles resulting in foot drop. The weakness is slowly progressive, eventually causing symmetric lower and upper extremity weakness, classically sparing the quadriceps muscles.

**Table 2:** Common features of GNE myopathy.

Clinical features	• Typical onset in 3rd decade of life • Initial symptom most often foot drop • Slow progression to upper extremities and proximal muscles • Spares quadriceps muscles • Not associated with cardiomyopathy • Respiratory dysfunction uncommon, may be seen in non-ambulatory patients
Genetics	• Autosomal recessive inheritance • Biallelic mutations in GNE gene • No clear genotype/phenotype correlation
Muscle pathology features	• Fiber size variation • Endomysial fibrosis • Rimmed vacuoles • Amyloid deposition • Non-inflammatory muscle biopsy • 14–18 nm filamentous inclusions on electron microscopy

Weakness in the upper extremities typically appears about 10 years after onset of symptoms in the legs, and most often presents as grip weakness. Though the distribution of weakness in the upper extremities may be variable, intrinsic hand muscles and deep finger flexors are typically most prominently affected. There may also be scapular weakness resembling a scapuloperoneal syndrome, though typically scapular weakness is seen only later in the disease course (32). Scapular winging is not uncommon, and was observed in 11 out of 26 patients in the North British cohort (21).

A common phenotypic feature is the preservation of the quadriceps muscles until late in the disease process. One observation made by Argov was that of a wheelchair-dependent patient in his 60s, extending his knees with his granddaughter sitting on his ankles (33). Often, this sparing of the quadriceps persists despite profound weakness resulting in loss of ambulation. This also seems to be quite consistent among nearly all cases, though the underlying cause of this is not known (34). It is notable that the quadriceps muscle contains similar amounts of GNE protein as other muscles (34). It is not known whether there may be some compensatory mechanism which is found in the quadriceps muscle that is not found in other muscles, though this has not been found. Regardless, the maintained strength in the quadriceps muscles allows for ambulation much later in the course of the disease than may be expected based on the extent of weakness of other muscles.

In general, respiratory function is not significantly affected in patients with GNE myopathy. Patients from the British and non-Jewish Iranian cohorts all had normal respiratory function (21, 22). Patients from the Bulgarian Roma cohort were reported to have some reduction of forced vital capacity (FVC), though overall respiratory function was relatively preserved. In this cohort, FVC was reduced to 60–75% in 5 out of 27 individuals. This may be related to disease severity, which was also observed in a natural history study in the Japanese cohort showing that FVC declines in non-ambulatory patients (35). In the Japanese cohort, 12 out of 39 patients had at least mild respiratory dysfunction with FVC <80%. 11 of the 12 patients were non-ambulatory. Respiratory dysfunction, if present tends to correlate with overall disease severity. In one case however, significant respiratory dysfunction was described in a patient who had a late onset of weakness in his 50s (36). Overall, the respiratory dysfunction is typically mild, and severe respiratory dysfunction is not typical, even with advanced progression of the disease.

Likewise, there does not appear to be significant cardiac involvement in GNE myopathy. The Bulgarian Roma cohort did have reports of cardiac abnormalities, though it is not clear whether these were related, and the majority were not clinically significant (15). These included changes seen on echocardiogram or electrocardiogram. However, cardiac involvement has been seen in a mouse model study. About 20% of Gne knockout mice expressing the GNE p.D207V mutation were found to have cardiac fibrosis. Amyloid deposition and occasional rimmed vacuoles were also seen in cardiac muscle tissue (37). These mice were also found to have similar findings in the diaphragm muscle.

Thrombocytopenia has recently been reported in patients with GNE myopathy in some East Asian patients. In 2014, Zhen et al. described two adult patients from a family who both had GNE myopathy and thrombocytopenia (38). In that same year, Izumi et al. separately reported two adult patients from one family who also had GNE myopathy and congenital thrombocytopenia. These patients were compound heterozygotes and the mutations were not the same between families (39). Additional cases have since been described, including two infant patients with congenital thrombocytopenia due to GNE mutations who may be pre-symptomatic for muscle disease.

## Diagnostic features

Patients presenting with distal weakness in a pattern and age of onset concerning for GNE myopathy may undergo testing including electromyography, genetic testing, and muscle biopsy. The diagnosis typically relies on a clinical presentation consistent with GNE myopathy as well as characteristic muscle pathology findings. Confirmation of the diagnosis is made by genetic testing showing pathogenic homozygous or compound heterozygous mutations affecting both alleles of the GNE gene. Advances in genetic testing over the past few decades, along with its availability and reliability have led to its widespread use as a diagnostic test.

Serum creatinine phosphokinase (CK) levels may be normal to mildly elevated (15, 20). This would be expected to decrease over time with disease progression, and the CK level in non-ambulatory patients may be normal or low (40).

Electromyography is expected to show myopathic features particularly in clinically affected muscles. Testing may show the presence of spontaneous activity such as fibrillation potentials and positive sharp waves indicative of muscle membrane irritability (21).

Muscle imaging is increasingly used in neuromuscular conditions. In the case of GNE myopathy, muscle imaging may reveal patterns of atrophy and can also be helpful for muscle biopsy site selection. Magnetic resonance imaging (MRI) findings typically demonstrate muscle atrophy and fatty replacement most significantly in the ankle dorsiflexors with sparing of the quadriceps muscles consistent with the clinical pattern of weakness. In one assessment of the imaging features of 13 GNE myopathy patients, several muscles appeared to be affected more than others. Even relatively early in the disease, severe involvement was seen in the biceps femoris short head to a greater degree than the distal muscles such as the tibialis anterior, extensor hallucis and digitorum longus, soleus and gastrocnemius muscles (41). Muscle ultrasonography has also been used to assess patients with GNE myopathy. In an analysis of six patients of Iranian-Jewish descent and homozygous for the p.M743T mutation all had a target-like appearance of the hamstring muscles with central atrophy and peripheral sparing (42).

Muscle biopsy shows several consistent features. In the description by Nonaka from 1981, muscle biopsy showed remarkable fiber size variation with endomysial fibrosis. Rimmed vacuoles were seen as well as atrophic muscle fibers. Electron microscopy showed the presence of intracytoplasmic vacuoles with concentric lamellar bodies and autophagic vacuoles (1). 14-18nm filamentous inclusions are also seen. Biopsy also demonstrates amyloid deposition with Congo-red staining. Vacuoles with strong acid phosphatase positivity and reactivity with lysosomal markers are also seen, indicating evidence of autophagic activity (25). Significant inflammatory infiltration is not expected in GNE myopathy. Biopsy of unaffected muscles typically does not show these characteristic findings. In one patient described by Nonaka, biopsy of the relatively spared vastus medialis muscle showed only minimal fiber size variation and only a few fibers with acid phosphatase positive cytoplasmic spots.

## Biomarkers

There has been research interest into a number of potential biomarkers for GNE myopathy. A reliable biomarker is of value both for the purposes of diagnosis as well as evaluating response to potential treatments, possibly reducing the need for serial muscle biopsies. Sialylation of blood-based glycans is one such biomarker that has been recently studied. Leoyklang et al. studied the sialylation of plasma O-glycan species in GNE myopathy patients and found that those with GNE myopathy had a significantly higher proportion of unsialylated vs. sialylated species in comparison to healthy controls (43). This was measured by assessing the unsialylated Thomsen-Friedenreich (T) antigens compared to the sialylated (ST) antigens. All GNE myopathy patients had an increased T/ST ratio.

Sialylation of other molecules has also been shown to be a potential diagnostic marker. Valles-Ayoub et al. looked at sialylation of neural crest cell adhesion molecules (NCAM), and found that NCAM was hyposialylated in patients with GNE myopathy (44). NCAMs are thought to play a role in myogenesis and neuromuscular development, making them a relevant target. One challenge in prior investigations is that the majority of serum proteins are secreted from hepatocytes and may not accurately represent sialylation of muscle-related components.

Muscle imaging has also been proposed as a potential biomarker. Liu et al. used MRI and proton maGNEtic resonance imaging (H-MRS) among GNE myopathy patients representing a wide range of disease progression (45). They found that quantitative and qualitative muscle imaging with MRI and H-MRS is a useful, non-invasive approach to characterize and monitor disease progression.

There may also be a potential use for lectins in quantifying sialylation levels. Lectins are proteins which bind sugars, and specific lectins bind specific carbohydrate sequences. Certain lectins bind to sialic acid. Others bind to desialylated carbohydrate sequences which could potentially be used to demonstrate hyposialylation in patients with GNE myopathy. Studies so far have focused on lectin characterization in muscle tissue *via* fluorescent staining and have shown promise as potential biomarkers (46, 47).

## Genetics

GNE myopathy follows an autosomal recessive inheritance pattern. The majority of affected individuals are compound heterozygotes, particularly in locations where there is not a single dominant mutation. 21 out of 26 patients in the British Isles cohort were found to be compound heterozygotes despite the presence of two commonly occurring mutations in that region. Pathogenic GNE mutations are most commonly missense mutations, though nonsense, insertions, deletions, intronic variants and splice site mutations have also been reported (21, 48). There have been over 200 reported pathogenic mutations in the GNE gene besides the known founder mutations (3). The majority of pathogenic GNE gene mutations are sporadic or involving a few families, though some mutations recur frequently in certain populations (40).

The GNE gene is located on chromosome 9p12-13 and contains 13 exons (40). There are at least eight GNE mRNA splice variants, the largest of which are referred to as the hGNE1 and hGNE2 isoforms. hGNE1 was the isoform initially described, and covers 722 amino acids. The hGNE2 isoform covers 753 amino acids (49). Therefore, there is a discrepancy of 31 amino acids between the previously accepted nomenclature and the updated nomenclature, as recommended by an international consortium on the subject in 2014. For example, the so-called Middle Eastern variant affecting the Iranian-Jewish population was previously referred to as the p.M712T variant, but is now known as the p.M743T variant. Likewise, the Japanese variant previously referred to as the p.V572L variant is now known as the p.V603L variant. This difference of 31 base pairs represents the discovery of the additional N-terminal sequence (50).

In mice, it has been observed that complete deletion of the GNE gene is not survivable (51), and no patients have been identified with complete gene knockout (52). This suggests that GNE plays an essential role in early development. GNE is active in the muscle, though rodent studies have shown that the highest GNE expression is in other tissues including liver, brain, lung, and kidney (53). Perhaps surprising considering the purported pathogenic mechanisms, these tissues are not affected in patients with GNE myopathy (21). GNE gene expression is found in high levels in immature myoblasts, with lower levels found in mature skeletal muscle (54). The expression of GNE is increased in damaged and regenerating muscle fibers. Though typically found in the cytoplasm, the GNE protein has been found to be significantly translocated from the cytoplasm to the nucleus in regenerating muscle fibers which may suggest a change in functioning of the GNE protein through the regeneration process (55).

As the GNE protein is bifunctional containing both kinase and epimerase functions, mutations may affect either of these domains. For example, the p.M743T mutation affects the kinase domain, and the p.D207V mutation affects the epimerase domain. Functionally however, an abnormality in one domain has been shown to also affect the other domain (31).

## Etiology

**Figure 1:**
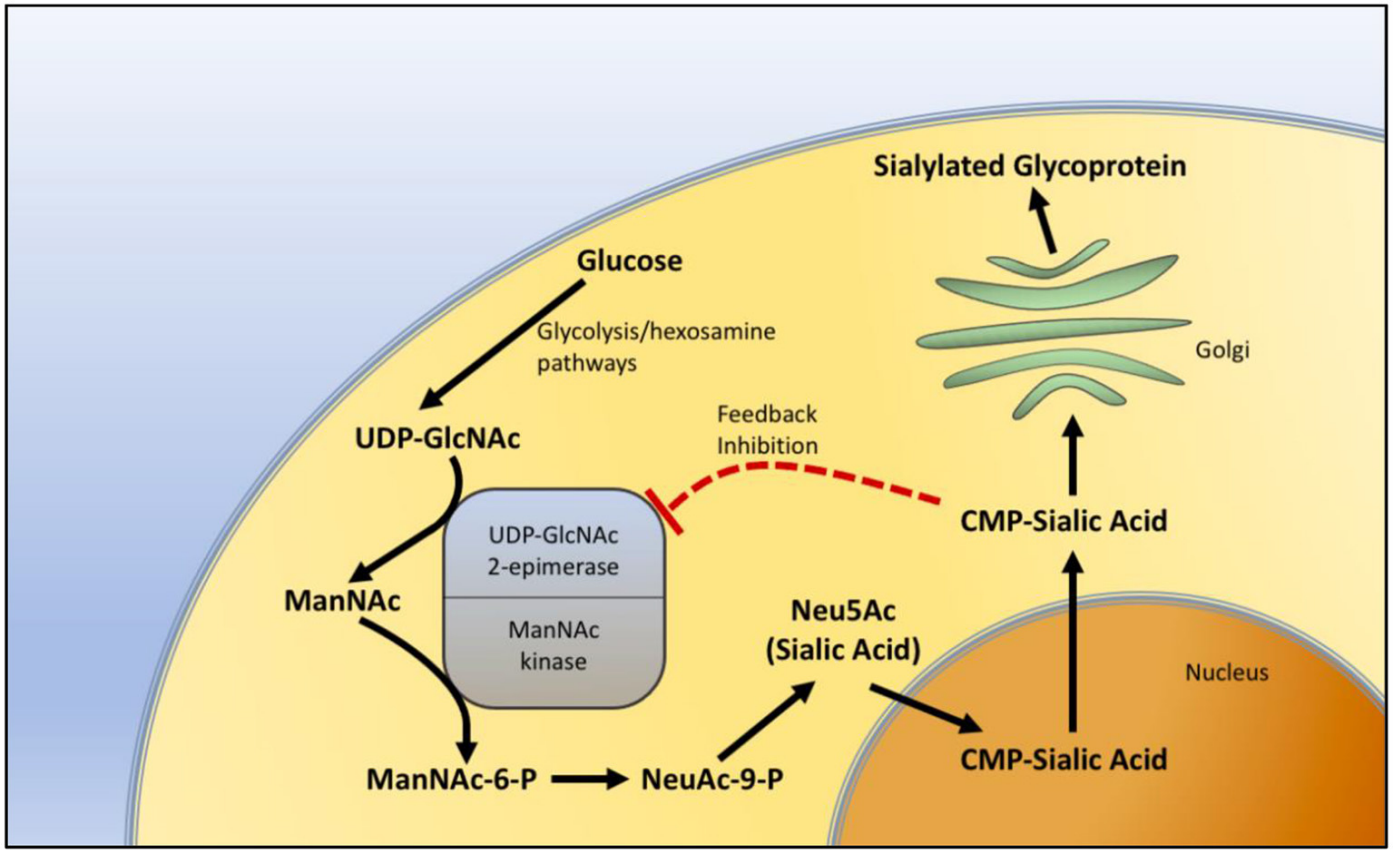
Sialic acid biosynthesis pathway. The biosynthesis of sialic acid in the cell. Uridine diphosphate N-acetylglucosamine (UDP-GlcNAc), a metabolite of the glycolytic and hexosamine pathways, is epimerized to N-Acetylmannosamine (ManNAc) and then phosphorylated to N-Acetylmannosamine-6-phosphate (ManNAc-6-P) by sequential components of the GNE enzyme, representing the two rate-limiting steps in the production of sialic acid. In the nucleus, sialic acid is further metabolized into the active form of sialic acid, cytidine-5'-monophosphate N-acetylneuraminic acid (CMP-sialic acid) and then added to glycoproteins in the Golgi during the process of sialylation. CMP-sialic acid also provides feedback inhibition of UDP-GlcNAc 2-epimerase.

GNE myopathy has been thought to be related to sialic acid. The GNE gene product plays an important role in the biosynthesis of sialic acid. It catalyzes the first two steps in the pathway of sialic acid production. The GNE gene encodes both UDP-N-acetylglucosamine (GlcNAc) and 2-epimerase/N-acetylmannosamine (ManNAc) kinase, constituting both portions of the enzyme. GlcNAc epimerase catalyzes the production of ManNac from UDP-GlcNAc, and ManNac kinase catalyzes the production of ManNAc-6-P from ManNac. These reactions represent the rate-limiting initial two steps in the production of N-acetylneuraminic acid (Neu5Ac), also known as sialic acid (Figure 1).

GNE protein levels may not directly correlate with disease. Krause et al., using immunofluorescence detection of the GNE protein, observed that the GNE protein is expressed at the same levels in GNE patients and it is in normal controls (56). Therefore, it is thought that the disorder may be more related to dysfunction of the GNE protein rather than the quantity. GNE is expressed in all tissues, though it remains unclear as to why skeletal muscle is affected and other tissues are not, though mouse models have shown significant disease in other tissues (57).

The relationship between sialic acid levels and GNE myopathy may not be a simple one either. Sialic acid has been found to be reduced in some studies. An early observation indicated reduction of sialic acid levels in one Japanese patient (58). Additional work on fibroblast and myocyte cultures of GNE patients showed the reduction to be as much as a 60–75% in comparison to normal (59). Sialylation appeared to be restored when cells were fed ManNAc or sialic acid. Salama et al. observed that myoblasts derived from patients with homozygous epimerase domain mutations resulted in significantly reduced membrane-bound sialic acid, though homozygous kinase domain or compound mutations did not have the same effect. This may indicate that dysfunction of either the epimerase or kinase component of the enzyme, though expected to have the same effect, may not equally affect the overall sialylation in muscle cells. Additionally, sialic acid levels in normal control subjects have a broad range. Sialic acid levels were assessed in cultured muscle fibers which showed overlap in levels between cells from affected individuals and normal controls (60).

## Role of sialic acid

Sialic acids are monosaccharides attached to the terminus of cell surface glucoconjugates, where they have an important role in cellular recognition and adhesion, regulate glycoprotein stability, and may function in wound healing (7, 51, 60). They also play a role in tumor formation, progression, and metastasis (61). They act as ligands for cellular receptors or other intrinsic protein receptors found in the body, as well as those of extrinsic viruses and other toxins (62). Sialic acids are involved in fertilization and embryogenesis, and though it is known that GNE gene knockout in mice is not survivable, the mechanism as to why this is the case is not known (51).

Sialic acid is also important for platelet function. Thrombocytopenia appears to be related to desialylation of platelets causing subsequent platelet destruction. Sørensen et al. demonstrated that this platelet clearance is a result of asialoglycoprotein receptor-expressing scavenger cells (63). This is the same mechanism which appears to cause thrombocytopenia in patients with sepsis. Under normal conditions, platelets gradually lose surface sialic acid over time as a result of sialidases. Interestingly, oseltamivir has been shown in case reports to increase platelet counts in patients with immune thrombocytopenia, likely as a result of its role as a sialidase inhibitor and thus decreasing the clearance of platelets (64). As detailed above, there is increasing evidence of a relationship between GNE myopathy and thrombocytopenia.

Sialic acid is also implicated in other disorders, though these result in an excess rather than a deficiency of sialic acid. One disorder of sialic acid production also associated with mutations in the GNE gene is known as sialuria. In normal conditions, the allosteric site of GNE epimerase is inhibited by sialic acid acting as a feedback inhibition mechanism. Sialuria is an autosomal dominant condition characterized by excess cytoplasmic accumulation of free sialic acid due to dysfunction of this feedback inhibition (65). This is a very rare disorder characterized by intellectual disability and hepatomegaly. The sialidoses are another group of disorders that result in the accumulation of intralysosomal sialic acid resulting from the inability to remove sialic acids from glucoconjugates by dysfunctional lysosomal sialidase (66).

It is not entirely clear if the disruption of the known functions of sialic acid explains the extent of muscle disease in GNE myopathy. As the role of sialic acid and how it relates to myopathy is uncertain, additional mechanisms have also been considered. Sialic acid plays a role in proliferation, so one consideration is that reduced sialic acid may induce muscle fiber apoptosis. Singh and Arya showed in a cell-based model that cells with GNE mutations had defective cell proliferation. These GNE mutant cells were also found to have abnormal mitochondrial structural changes and transmembrane potential alterations suggesting a mitochondria-dependent cell apoptosis mechanism (67).

GNE myopathy is also known to be associated with the accumulation of amyloid β-peptide (Aβ) in muscle cells. Bosch-Morató et al. proposed a disease mechanism where amyloidosis may be the result of hyposialylation, ultimately resulting in muscle fiber apoptosis in GNE myopathy (52). Hyposialylated myoblasts were found to have significantly increased uptake of extracellular Aβ1-42. The Aβ1-42 uptake was subsequently prevented when these cells were resialylated. The mechanism of amyloid β-peptide endocytosis was found to be clathrin based when due to hyposialylation, as opposed to caveolin based, another known mechanism of amyloid β-peptide endocytosis.

## Treatment trials

There have been a number of therapeutic trials in GNE myopathy, though there remains no approved treatment at this time. Trials have focused on increasing sialic acid as well as immunomodulatory therapy.

A trial looking at the efficacy of IVIg in 2007 showed mixed results. In this case, IVIg was investigated for its potential to provide sialic acid (68). Four patients were treated with IVIg with a loading dose followed by 3 weekly maintenance infusions. There were some gains noted in strength, though objective testing was less definitive. For example, grip strength improved an average of 5% by the end of the study, matching the improvement seen in tongue strength, which is not thought to be affected in GNE myopathy. Additionally, muscle biopsy did not show evidence of increased sialylation in muscle tissue after the course of IVIg.

Other potential therapies have focused on the production of sialic acid. Results from mouse studies have indicated that ManNAc may be a promising treatment. One such study by Galeano et al. published in 2007 included mice homozygous for the M743T mutation. Mice given oral ManNAc showed increased survival as well as increased GNE protein expression, as well as increased sialylation of NCAM and podocalyxin (69). Another mouse model study in 2009 showed significant improvement in strength, prevention of atrophy, reduction in amyloid deposits and rimmed vacuoles among other markers (70). Additionally, it was shown that administration of a precursor of ManNAc known as peracetylated ManNac, or tetra-*O*-acetyl-*N*-acetylmannosamine (Ac_4_ManNAc) increased sialylation and improved strength in mice. Sialyllactose has also been studied for use as a potential treatment and was included in this study as well and produced similar results. Sialyllactose is a conjugated sialic acid which is broken down in the lysosome to release free sialic acid. Further mouse model studies reinforced these results, showing increased sialic acid levels in the blood, as well as recovery of muscle size to the levels of wild type mice and improvement of rimmed vacuoles and amyloid on biopsy (71).

Sialic acid and its precursors then became a focus of potential therapies in humans. A phase 2 clinical trial in 2012 investigated the use of a sialic acid precursor in patients with GNE myopathy. An extended-release form of free sialic acid N-Acetylneuraminic acid (Neu5Ac) was used due to the rapid clearance of free sialic acid. Administration of this extended-release form appeared to demonstrate significant improvement in upper extremity composite strength (72). However, the subsequent phase 3 trial did not reproduce these findings (73).

ManNAc is being studied for use in humans with GNE myopathy. The phase 1 ManNAc trial showed a prolonged increase in plasma Neu5Ac levels (74). This may seem unexpected in consideration of the known sialic acid biosynthesis pathway in which ManNAc must be further catalyzed by ManNAc kinase, the very enzyme known to be dysfunctional in GNE myopathy. This increase in Neu5Ac was even found among patients homozygous for kinase domain mutations. This may be at least in part related to the action of GlcNAc kinase having some ability to phosphorylate ManNAc. Also, it appears that the ManNAc given by supplementation is not affected by feedback inhibition.

The phase 2 clinical trial of ManNAc in GNE myopathy started in 2015 and published in 2021. This trial demonstrated safety and tolerability of ManNAc, as well as indicating a slower rate of decline in both upper and lower extremity strength, as well as increased plasma sialic acid (Neu5Ac) and sarcolemmal sialylation (75). A multi-center study of ManNAc for GNE myopathy has just started (NCT04231266).

As GNE myopathy is a genetic condition, gene therapy has been an area of interest as a potential treatment. The GNE gene was prepared as a lipoplex and administered to one patient intravenously every 60 days for a total of 7 doses. It appeared to be well tolerated, and appeared to show stability or modest improvement (76). Aside from this single case of compassionate use of an investigational treatment, gene therapy has not been investigated in GNE myopathy.

## Future directions

Some aspects of GNE myopathy may benefit from further study (Table 3). Certain aspects relating to pathogenesis and disease characteristics may still be of value to our understanding of the disease and for potential treatments. For instance, the animal models of GNEM do not have a reliable muscle phenotype. The knock-in mouse models showed glomerular disease that is not seen in human models as well as neonatal death unless treated with sialic acid increasing therapy (69). *Gne*^(−/−)^h*GNE*D207V-transgenic mice showed early involvement of the gastrocnemius and quadriceps muscles in mice which is usually a late finding in humans (70).

**Table 3:** Areas for future study—Unmet needs in GNE myopathy.

• Lack of phenotypically appropriate animal model • Reliable antibodies to GNE protein not available • Doubts about whether hyposialylation is the causative mechanism • Lack of reliable biomarkers (MRI vs. lectin staining vs. biochemical serum markers) • Resolution of common and difficult to interpret variants of unknown significance • Need for better assays to measure GNE enzymatic function

There is a dearth of validated antibodies to GNE to help detection of the protein in tissues. The reasons underlying the relative sparing of the quadriceps muscles remains unknown. If the factors responsible for this sparing could be identified, then perhaps these factors may give insight into potential treatments. The role of sialic acid in disease pathogenesis is still not entirely clear. At the least, there does not appear to be a direct correlation of muscle disease with GNE gene expression or sialic acid levels. There may be more complex factors involved.

The search for adequate biomarkers is currently an area of ongoing study. Reliable biomarkers are essential in determining response to a potential treatment. GNE myopathy trials have often relied on motor strength assessments, muscle biopsy features, and patient-reported outcomes. An appropriate biomarker may ideally allow for a more objective response to treatment monitoring and may also aid in diagnosis.

Gene therapy remains another area of interest. This mode of treatment does have risks however, including immune responses and malignancy (77). The death of a participant in a gene therapy trial for ornithine transcarbamylase deficiency in 1999 due to immune response, as well as the uncertainty regarding these treatments resulted in a cautious approach for many years. The Food and Drug Administration has only recently approved gene therapy treatments for other conditions since 2017. Recombinant adeno-associated viruses (rAAV) have often been studied as gene delivery vectors. AAV8 was found in a mouse model study to be able to deliver the GNE gene to muscle cells and appeared safe and efficient (78). It should be noted that a significant number of people have antibodies to AAV8 (79). There were four recent unfortunate deaths in a trial using the AAV8 vector for gene delivery in myotubular myopathy (80, 81). At least three of these deaths appear to be related to liver dysfunction. Other AAV vectors are being used in gene therapy treatment for Duchenne muscular dystrophy and spinal muscular atrophy.

## Conclusion

From the initial descriptions of what would be later named GNE myopathy, to the genetic characterization and more recent treatment trials, there has been substantial advancement in the understanding of this disorder. Despite these advances however, unanswered questions remain. As of yet there is no approved disease-modifying treatment. Management continues to focus on supportive therapies. Several features of this disorder have not yet been fully explained, including why the quadriceps and other organs are spared despite the ubiquity of the GNE gene, as well as a full understanding of the role of sialic acid in the disease process. GNE myopathy is still likely underdiagnosed. Improved awareness and better access to appropriate testing is likely to identify more affected patients. Ideally, as more is understood about this disorder over time, a viable treatment may be found.

## Author contributions

JM, KA, and TM contributed to conception and design of the study and wrote sections of the manuscript. JM and KA performed the literature search and wrote the first draft of the manuscript. All authors contributed to manuscript revision, read, and approved the submitted version.

## Conflict of interest

Author TM discloses an advisory role for and/or receiving research funds from Alexion, Amicus, Argenx, Arvinas, Audentes, AvroBio, Horizon Therapeutics, Immunovant, Maze Therapeutics, Momenta now Janssen, Sanofi-Genzyme, Sarepta, Spark Therapeutics, UCB, and Modis/Zogenix and also serves on the data safety monitoring board for Acceleron, Avexis, and Sarepta. The remaining authors declare that the research was conducted in the absence of any commercial or financial relationships that could be construed as a potential conflict of interest.

## Publisher's note

All claims expressed in this article are solely those of the authors and do not necessarily represent those of their affiliated organizations, or those of the publisher, the editors and the reviewers. Any product that may be evaluated in this article, or claim that may be made by its manufacturer, is not guaranteed or endorsed by the publisher.
